# Data on rare earth elements in different particle size fractions of topsoil for two small erosional landforms in central European Russia

**DOI:** 10.1016/j.dib.2020.105450

**Published:** 2020-03-18

**Authors:** Оlga A. Samonova, Elena N. Aseyeva, Olga V. Chernitsova

**Affiliations:** a Independent researcher; b Lomonosov Moscow State University, Faculty of Geography, GSP-1, Leninskie Gory, 119991, Moscow, Russia

**Keywords:** Rare earth elements, Topsoil, Particle size fractions, Forest zone, Small erosional landforms, Lateral distribution

## Abstract

The pathways and behavior of rare earth elements (REEs) in the soil environment have been receiving greater significance due to their wide use in technological applications, agriculture, and medicine over the last two decades and insufficient information on their health effect and participation in soil and geochemical processes. In this paper, we report original data on rare earth elements in various particle size fractions separated from the topsoil horizons of two small erosional landforms located in an uncontaminated area of the central part of European Russia (the Middle Protva basin, the Kaluga region). Soil samples were collected from the top 10 cm along several soil transects. Soils were sampled at the landforms sides, bottoms, detrital fans and catchment areas considered as sources of solid matter. The sampling scheme used makes it possible to assess the REEs distribution from catchments to bottoms of the erosional landforms, as well as along their thalwegs. The collected bulk samples (*n* = 22) were physically fractionated and the concentrations of Sc, Y, La, Ce, Pr, Nd, Sm, Eu, Gd, Tb, Dy, Ho, Er, Tm, Yb, Lu were determined in five particle size fractions (1000–250, 250–50, 50–10, 10–1 and <1 µm, *n* = 100) by ICP-MS using Elan-6100 spectrometer (Perkin Elmer Inc., USA). The data obtained also include the concentrations of Fe and Mn (ICP-AES), as well as the information on the total content of organic carbon (TOC), pH and particle size distribution of the bulk samples. The obtained dataset can be used for various purposes: it is suitable as a baseline for the assessment of pollution levels, exploring natural and anthropogenic anomalies, for revealing the association of REEs with specific particle size fractions and detecting the effect of parent material and lateral translocations of soil material and soil particles on the REEs levels.

Specifications table

**Table:** 

Subject	Earth Science, Environmental Chemistry		
Specific subject area	Earth Science, Environmental Chemistry, Soil Chemistry, Landscape Geochemistry, Rare Earth Elements in Soil Environment		
Type of data	Tables with raw data; Figures		
How data were acquired	Inductively Coupled Plasma-Mass Spectrometry (ICP-MS) and Inductively Coupled Plasma Atomic Emission Spectrometry (ICP-AES). Models: Elan-6100 ICP-MS System (Inductively Coupled Plasma Mass Spectrometer by Perkin Elmer Inc., USA); Optima-4300 DV ICP-AES System (Inductively Coupled Plasma Atomic Emission Spectrometer by Perkin Elmer Inc., USA). Data on physicochemical properties of soils were obtained by means of standard techniques [1,2]		
Data format	Raw		
Parameters for data collection	Samples were collected in an uncontaminated area from topsoil horizons of two small erosional landforms that have different morphology and belong to different lithological types. Samples were taken of the top 10 cm at 22 locations (11 locations in each landform).		
Description of data collection	The sample collection was performed of the top 10 cm along several soil transects crossing the upper, the middle and the lower reaches of the landforms and also along their thalwegs. Four sets of soil samples were obtained in each landform. They include the upper soil horizons of (1) the landform sides, (2) the bottom and (3) the detrital fan and also (4) the adjacent catchment area considered as a source of solid matter. The collected bulk samples were sieved, dried and used for physical fractionation into five particle size fractions and physicochemical analyses using conventional methods. For determining concentrations of REEs and Mn, Fe the bulk samples and soil particle size fractions were analysed by ICP-MS and ICP-AES after digestion in a mixture of HCl, HNO_3_, HF, and HClO_4_ acids (NSAM-499-AES/MS method).		
Data source location	The sampling sites were located in the south-eastern part of the Smolensk-Moscow Upland, 100 km to the southwest from Moscow (Russia). GPS coordinates for the sampling locations were as follows:		
	G-c-1	55° 12' 42,9"N	36° 20' 34,91" E
	G-c-2	55° 12' 41,83" N	36° 20' 34,07" E
	G-s-3	55° 12' 41,83" N	36° 20' 34,69" E
	G-b-4	55° 12' 41,84" N	36° 20' 35,16" E
	G-c-5	55° 12' 39,21" N	36° 20' 33,25" E
	G-s-6	55° 12' 39,19" N	36° 20' 33,98" E
	G-b-7	55° 12' 39,23" N	36° 20' 35,28" E
	G-c-8	55° 12' 36,78" N	36° 20' 34,5" E
	G-s-9	55° 12' 36,77" N	36° 20' 35,12" E
	G-b-10	55° 12' 36,8" N	36° 20' 35,52" E
	G-f-11	55° 12' 35,37" N	36° 20' 35,75" E
	B-c-12	55° 13' 6,2" N	36° 22' 24,07" E
	B-c-13	55° 13' 5,0" N	36° 22' 23,84" E
	B-s-14	55° 13' 5,0" N	36° 22' 24,5" E
	B-b-15	55° 13' 5,0" N	36° 22' 25,0" E
	B-s-16	55° 13' 1,48" N	36° 22' 23,23" E
	B-b-17	55° 13' 1,41" N	36° 22' 23,67" E
	B-c-18	55° 12' 58,93" N	36° 22' 20,85" E
	B-s-19	55° 12' 58,88" N	36° 22' 21,48" E
	B-b-20	55° 12' 58,8" N	36° 22' 22,11" E
	B-b-21	55° 12' 55,58" N	36° 22' 21,56" E
	B-f-22	55° 12' 52,76" N	36° 22' 18,66" E
Data accessibility	Data are with this article		

## Value of the data

• These are first open access data on levels of REEs in soils and soil particle size fractions for central European Russia. These data provide baseline and can be used in the assessment of pollution levels in a contaminated (for example, in urban) environment.
• The data can be used in statistical analysis and contribute to a better understanding of the relationship between the REEs contents in bulk soil material and its physicochemical properties (particle size distribution, organic carbon content, pH, Fe and Mn levels).
• The information on the partitioning of REEs among soil particle size fractions is very scarce. Our data will help to fill the data gap and are useful to evaluate the possible contribution of each particle size fraction to the total content of REEs and to reveal the most REE-rich fraction. This might be helpful in the geochemical assessment of natural and technogenic REEs anomalies.
• These data can be used by any researcher who undertakes a study on environmental behavior and pathways of REEs. The dataset can be used to detect the participation of REEs in geochemical processes, and to determine the factors controlling the partitioning of REEs among various particle sizes, such as parent material and lateral translocations of soil material.

## 1 Data description

**Fig. 1:**
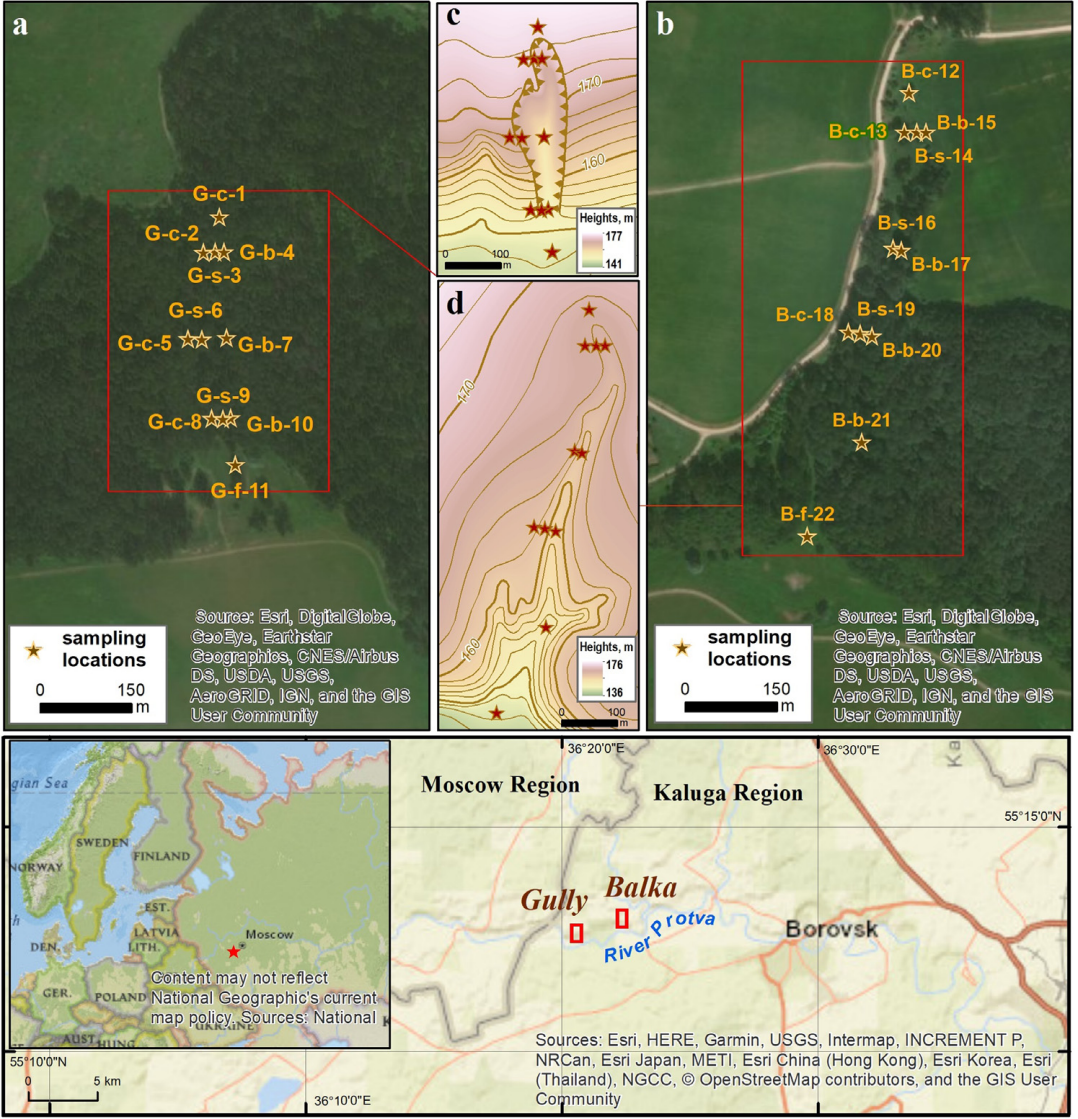
Sampling sites; sampling locations in the gully (a) and in the balka (b); topography of the gully (c) and the balka (d). Denotations in sampling location names are as follows: G – gully, B – balka, c – catchment, s – side, b – bottom, f – fan.

**Fig. 2:**
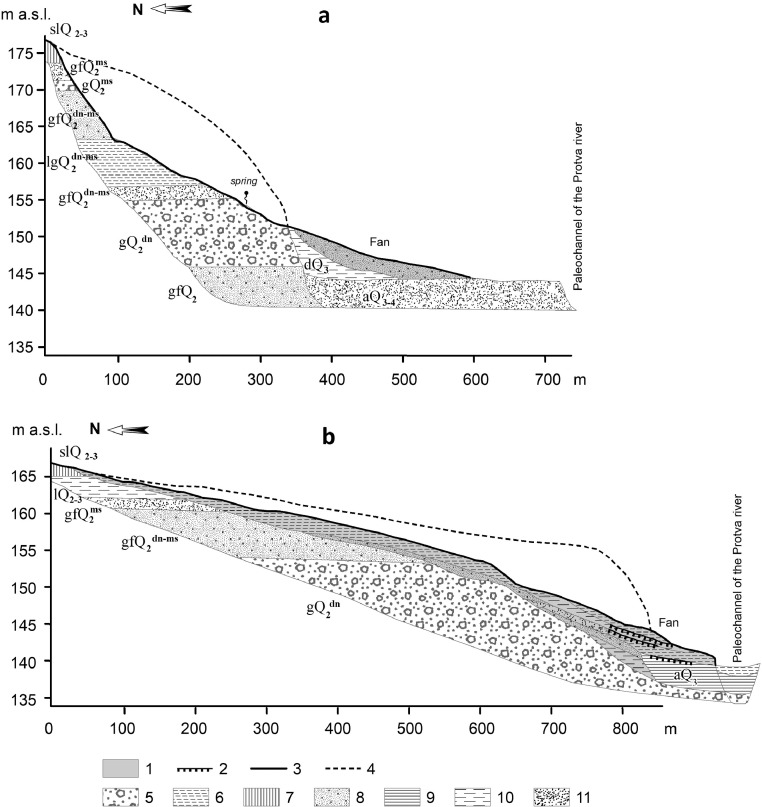
Longitudinal profiles of the gully (a) and the balka (b) incised in different types of Quaternary deposits (adopted from [10]). 1 – gully sediments, 2 – buried soils, 3 – gully thalweg and fan surface, 4 – gully edge, 5 – boulder loam (till), 6 – silt, 7 – mantle (loessial) loam, 8 – sand with gravel, 9 – clay, 10 – loam, 11 – sand. Geological indexes: Origin: a – alluvium, sl – mantle loam, g – glacial till, gf – glaciofluvial, lg – limno-glacial; Age: Q_2_ – Middle Pleistocene, dn – Dnieper stage (MIS-8), ms – Moscow stage (MIS-6), Q_3_– Late Pleistocene, Q_4_ – Holocene.

Fig. 1 displays soil sampling locations, as well as the topography of the erosional landforms. The number of each location is supplemented by a capital letter “G” or “B” (“G” for gully and “B” for balka) and a low-case letter (c, s, b, f) indicating the position of a soil in the relief: c – for catchment, s – for landform side, b – for bottom and f – for fan. The longitudinal profiles of the two landforms showing parent material and underlying lithologies are presented in Fig. 2. Table 1 contains data on some physicochemical properties of topsoil horizons that might control the levels of REEs in bulk soil [3,^4^,5]: pH_KCl_, total organic carbon (TOC), clay content and contents of other particle-size fractions. Concentrations of the REEs (Sc, Y, La, Ce, Pr, Nd, Sm, Eu, Gd, Tb, Dy, Ho, Er, Tm, Yb, Lu) are given in Table 2 both for bulk soil samples and five particle size fractions (1000–250, 250–50, 50–10, 10–1 and <1 µm). Table 2 also gives the concentrations of Mn and Fe, since some literature sources have reported that their oxides have the greatest adsorption capacity [3,^4^,6]. The last section of Table 2 specifies the methods used for the determination of the elements and gives the information on the element detections limits.

**Table 1:** Physicochemical properties of topsoil (bulk samples).

Location	рН КСl	TOC%	Moisture content,%	Particle size fractions,%						
				1000–250 µm	250–50 µm	50–10 µm	10–5 µm	5–1 µm	<1 µm	∑<10 µm
G-c-1	5.9	0.8	1.38	2.55	1.08	60.11	12.49	10.39	13.38	36.26
G-c-2	5.8	2.4	1.45	3.87	1.96	58.94	10.71	11.45	13.07	35.23
G-c-5	7.9	2.1	1.26	2.51	49.77	26.74	5.18	7.62	8.18	20.98
G-c-8	6.5	1.6	1.24	7.76	5.89	56.54	7.21	11.02	11.58	29.81
G-s-3	6.8	3.2	2.25	14.35	19.28	31.59	7.44	9.42	17.92	34.78
G-s-6	7.9	1.9	1.53	11.32	40.75	25.91	4.96	7.96	9.1	22.02
G-s-9	5.6	3.3	1.48	20.7	17.26	32.32	7.55	9.83	12.34	29.72
G-b-4	6.2	4.1	2.26	20.42	21.88	33.15	9.9	4.01	10.64	24.55
G-b-7	6.7	1.9	1.33	21.16	20.79	37.78	5.76	6.16	8.35	20.27
G-b-10	7.1	2.1	1.25	23.64	23.86	32.25	5.34	6.89	8.02	20.25
G-f-11	7.2	1.9	1.06	31.83	23.21	25.80	5.50	2.81	10.75	19.06
B-c-12	6.0	2.5	1.56	0.99	0.92	61.28	13.89	13.01	9.91	36.81
B-c-13	6.0	1.9	1.01	1.25	0.43	64.94	14.87	11.16	7.35	33.38
B-c-18	6.2	3.9	0.97	2.8	3.63	61.66	12.04	9.21	10.66	31.91
B-s-14	6.1	2.2	2.43	2.28	1.54	65.68	13.94	9.75	6.81	30.50
B-s-16	6.3	1.2	1.23	4.81	4.80	55.56	12.56	11.17	11.10	34.83
B-s-19	6.1	4.1	2.16	16.05	13.96	35.4	7.93	12.35	14.31	34.59
B-b-15	6.1	2.6	1.44	0.87	1.08	63.15	13.8	11.36	9.74	34.90
B-b-17	6.5	1.2	1.19	0.63	1.65	62.5	12.23	12.46	10.53	35.22
B-b-20	6.0	2.5	1.95	2.03	0.71	62.83	11.09	12.08	11.26	34.43
B-b-21	6.3	0.6	1.57	9.42	13.53	46.98	6.42	11.3	12.35	30.07
B-f-22	6.3	0.7	1.71	2.72	6.28	61.86	10.42	10.91	7.81	29.14

**Table 2:** Concentrations of rare earth elements (mg/kg), iron (%) and manganese (mg/kg) in topsoil (bulk samples and particle size fractions).

	Fe	Mn	Sc	Y	La	Ce	Pr	Nd	Sm	Eu	Gd	Tb	Dy	Ho	Er	Tm	Yb	Lu
Bulk sample																		
G-c-1	2.4	1780.2	9.20	17.3	37.1	60	8.99	32.2	6.07	1.14	5.44	0.79	4.35	0.82	2.26	0.34	2.36	0.39
G-c-2	2.3	1161.0	8.76	17.9	31.8	71.8	7.34	26.8	4.77	0.94	4.53	0.65	3.64	0.66	1.92	0.31	2.00	0.33
G-c-5	2.1	379.3	5.85	13.6	22.0	47.3	5.45	20.3	3.97	0.81	3.74	0.54	3.06	0.60	1.66	0.23	1.68	0.25
G-c-8	2.2	665.6	8.41	18.8	32.9	71.8	7.73	28.3	5.28	0.98	4.70	0.69	3.82	0.72	2.02	0.32	2.10	0.33
G-s-3	3.1	851.4	8.59	16.8	27.9	60.4	6.91	25.0	4.88	1.05	4.70	0.68	3.84	0.68	2.05	0.30	1.86	0.30
G-s-6	2.3	456.7	6.72	18.1	25.2	54.4	6.42	24.0	4.77	0.98	4.49	0.67	3.66	0.64	1.88	0.28	1.81	0.28
G-s-9	2.6	410.2	7.06	13.2	27.3	58.8	6.57	23.9	4.41	0.88	3.99	0.61	3.19	0.61	1.65	0.25	1.68	0.26
G-b-4	2.3	727.6	6.96	14.3	25.6	54.8	6.39	23.3	4.46	0.88	4.10	0.59	3.36	0.62	1.75	0.26	1.71	0.26
G-b-7	2.3	549.5	6.73	14.0	24.4	53.6	5.96	22.1	4.25	0.89	3.77	0.57	3.14	0.60	1.68	0.25	1.63	0.27
G-b-10	2.1	348.3	6.10	13.8	22.8	49.3	5.55	21.7	4.19	0.88	3.79	0.57	3.14	0.60	1.71	0.25	1.74	0.27
G-f-11	2.3	441.2	6.10	13.9	23.6	50.8	5.80	22.3	4.19	0.94	4.08	0.58	3.25	0.62	1.65	0.25	1.63	0.25
B-c-12	2.4	928.8	9.22	17.8	33.9	53.8	8.03	29.1	5.54	1.08	4.91	0.70	3.93	0.74	2.12	0.32	2.23	0.34
B-c-13	2.6	1702.8	8.38	16.5	31.7	71.8	7.56	27.6	5.13	1.05	4.42	0.66	3.67	0.68	1.97	0.31	1.96	0.32
B-c-18	2.7	1161.0	7.53	17.1	30.2	68.8	7.44	27.4	5.10	0.98	4.50	0.68	3.73	0.67	2.03	0.33	2.14	0.33
B-s-14	2.9	1238.4	7.32	13.4	28.5	63.5	6.76	24.8	4.58	0.87	4.14	0.61	3.32	0.61	1.86	0.28	1.99	0.30
B-s-16	2.2	1006.2	7.75	15.9	29.1	65.2	6.79	25.3	4.71	0.91	4.15	0.62	3.53	0.67	1.91	0.29	2.05	0.30
B-s-19	2.5	596.0	7.99	14.7	27.6	61.6	6.24	23.6	4.34	0.85	3.83	0.56	3.19	0.59	1.67	0.27	1.71	0.28
B-b-15	2.4	1006.2	9.04	18.4	32.7	72.3	7.73	28.6	5.39	1.03	4.70	0.70	4.09	0.77	2.13	0.34	2.29	0.35
B-b-17	2.6	928.8	8.88	17.4	31.8	69.5	7.34	28.1	5.11	0.99	4.54	0.69	3.86	0.71	2.02	0.32	2.10	0.33
B-b-20	2.9	1548.0	8.63	18.5	34.8	56.6	8.41	31.8	6.02	1.08	5.13	0.74	4.30	0.75	2.24	0.33	2.31	0.37
B-b-21	2.2	1238.4	7.26	14.9	30.3	71.6	7.52	27.6	5.20	0.98	4.43	0.67	3.87	0.69	1.95	0.29	2.03	0.31
B-f-22	2.4	1238.4	7.81	17.6	32.0	72.5	7.57	28.1	5.28	1.04	4.51	0.69	3.84	0.73	2.05	0.31	2.22	0.34
Particle size fraction 1: 1000–250 µm																		
G-c-8	6.7	479.9	1.23	3.67	7.69	16.5	1.67	6.39	1.15	0.37	1.11	0.16	0.94	0.17	0.47	0.07	0.47	0.06
G-s-3	4.6	479.9	1.59	5.97	8.74	17.3	2.17	8.51	1.64	0.48	1.64	0.23	1.45	0.27	0.78	0.10	0.72	0.10
G-s-6	4.1	332.8	1.47	4.94	7.06	13.5	1.66	6.43	1.24	0.39	1.22	0.18	1.01	0.20	0.60	0.09	0.57	0.09
G-s-9	5.7	379.3	2.07	4.92	9.67	20.0	2.22	8.66	1.58	0.47	1.52	0.23	1.26	0.24	0.67	0.10	0.64	0.10
G-b-4	5.1	565.0	1.94	6.02	9.93	20.1	2.38	9.23	1.85	0.46	1.71	0.25	1.47	0.27	0.74	0.11	0.69	0.11
G-b-7	2.3	379.3	1.65	6.26	12.10	26.1	2.88	11.10	2.16	0.49	2.00	0.29	1.52	0.27	0.75	0.10	0.66	0.11
G-b-10	3.7	232.2	1.85	5.48	9.39	17.8	2.25	9.19	1.71	0.49	1.62	0.25	1.31	0.24	0.71	0.10	0.64	0.10
G-f-11	4.6	294.1	1.80	5.41	9.63	19.7	2.32	9.09	1.76	0.50	1.71	0.24	1.35	0.25	0.69	0.10	0.66	0.10
B-c-13	7.8	3018.6	3.53	7.89	14.80	45.7	3.43	13.70	2.56	0.57	2.41	0.36	1.94	0.36	1.04	0.16	1.01	0.16
B-c-18	3.9	3947.4	2.98	8.25	15.30	59.3	3.62	13.70	2.59	0.64	2.41	0.37	1.92	0.39	1.04	0.15	0.92	0.15
B-s-14	6.3	5418.0	2.74	6.78	13.50	55.3	3.11	11.80	2.20	0.60	2.08	0.32	1.74	0.31	0.92	0.13	0.90	0.13
B-s-16	2.1	2322.0	1.92	4.56	10.20	37.4	2.30	8.76	1.63	0.46	1.45	0.21	1.13	0.22	0.64	0.08	0.65	0.11
B-s-19	2.2	387.0	1.50	5.90	7.11	17.5	1.56	6.17	1.17	0.39	1.04	0.18	1.24	0.29	0.79	0.13	0.72	0.10
B-b-20	9.4	5959.8	2.86	7.75	15.90	73.9	3.76	14.60	2.83	0.67	2.59	0.41	2.01	0.37	1.02	0.14	0.98	0.15
B-b-21	2.1	2167.2	1.79	4.79	10.10	39.9	2.32	9.07	1.70	0.50	1.61	0.25	1.27	0.24	0.66	0.10	0.64	0.10
B-f-22	2.8	4411.8	4.06	8.72	19.90	74.9	4.60	16.80	3.33	0.76	2.94	0.44	2.38	0.45	1.16	0.17	1.19	0.18
Particle size fraction 2: 250–50 µm																		
G-c-1	7.3	588.2	2.57	7.50	15.10	31.1	3.58	13.80	2.53	0.44	2.27	0.33	1.90	0.36	1.07	0.17	1.14	0.18
G-c-5	1.3	193.5	2.10	8.53	15.90	33.2	3.91	14.80	2.85	0.56	2.61	0.37	2.11	0.39	1.10	0.16	1.09	0.17
G-c-8	6.6	387.0	2.65	6.55	10.70	22.4	2.59	10.20	1.88	0.45	1.80	0.28	1.62	0.31	0.95	0.15	1.08	0.17
G-s-3	1.1	147.1	2.02	5.88	27.90	51.4	5.31	17.70	2.78	0.43	2.18	0.31	1.67	0.28	0.73	0.11	0.79	0.12
G-s-6	2.1	216.7	2.15	8.20	13.10	26.7	3.28	12.60	2.51	0.52	2.22	0.32	1.92	0.36	1.05	0.16	1.01	0.16
G-s-9	2.6	201.2	2.36	5.91	16.10	33.4	3.87	14.50	2.72	0.40	2.08	0.31	1.58	0.30	0.86	0.14	1.05	0.16
G-b-4	3.6	294.1	2.91	6.53	12.30	25.2	2.94	11.30	2.05	0.43	1.88	0.28	1.54	0.29	0.87	0.14	1.23	0.15
G-b-7	2.0	216.7	2.03	5.85	10.80	22.0	2.50	9.50	1.81	0.39	1.60	0.24	1.36	0.27	0.78	0.12	0.86	0.13
G-b-10	3.2	247.7	3.17	8.90	17.70	36.7	4.30	17.30	3.02	0.62	2.91	0.42	2.25	0.44	1.24	0.20	1.38	0.21
G-f-11	2.8	178.0	2.44	5.80	9.96	20.7	2.36	9.30	1.71	0.42	1.55	0.23	1.27	0.26	0.74	0.12	0.82	0.13
B-c-12	5.2	394.7	2.53	11.50	15.60	32.1	3.62	12.40	2.51	0.48	2.28	0.38	2.02	0.40	1.37	0.22	1.50	0.23
B-c-13	8.7	611.5	2.31	9.53	14.70	30.8	3.60	13.30	2.71	0.44	2.24	0.34	1.85	0.41	1.16	0.19	1.24	0.19
B-c-18	9.2	735.3	2.59	8.47	19.90	41.4	4.54	17.00	3.14	0.62	2.71	0.38	2.24	0.38	1.20	0.18	1.20	0.19
B-s-14	4.5	510.8	2.36	10.90	16.60	35.8	4.00	13.70	2.84	0.48	2.46	0.39	1.98	0.40	1.29	0.21	1.40	0.31
B-s-16	4.1	371.5	2.20	8.36	13.90	28.7	3.13	11.40	2.12	0.42	1.89	0.29	1.73	0.32	0.93	0.16	1.14	0.18
B-s-19	4.5	332.8	1.71	5.29	7.63	15.2	1.67	6.16	1.37	0.34	0.99	0.17	0.95	0.19	0.61	0.11	0.70	0.10
B-b-15	8.7	626.9	2.47	9.72	16.00	32.5	3.70	14.00	2.79	0.48	2.23	0.35	2.09	0.42	1.14	0.18	1.25	0.20
B-b-17	8.9	712.1	3.26	12.50	18.10	40.6	4.32	17.50	3.48	0.65	2.67	0.38	2.62	0.55	1.65	0.30	1.60	0.24
B-b-20	4.7	603.7	2.98	12.70	19.90	44.1	4.82	19.50	3.68	0.61	2.99	0.45	2.76	0.55	1.61	0.25	1.59	0.25
B-b-21	5.9	510.8	1.53	4.88	7.30	15.4	1.69	6.08	1.22	0.34	1.09	0.18	1.07	0.20	0.65	0.09	0.64	0.11
B-f-22	2.5	278.6	2.13	7.73	13.30	29.0	3.17	12.20	2.19	0.45	2.03	0.28	1.63	0.27	0.88	0.15	0.94	0.15
Particle size fraction 3: 50–10 µm																		
G-c-1	1.40	263.2	5.00	15.7	28.3	58.1	6.66	24.9	4.76	0.86	4.23	0.63	3.62	0.68	2.03	0.31	2.20	0.35
G-c-2	1.61	247.7	4.70	16.2	28.0	58.9	6.77	25.3	4.82	0.84	4.18	0.64	3.66	0.70	1.98	0.31	2.17	0.33
G-c-5	1.50	278.6	4.83	18.7	23.2	49.8	5.95	22.7	4.50	0.88	4.19	0.61	3.61	0.71	2.08	0.31	2.07	0.33
G-c-8	1.94	224.5	5.33	14.8	31.5	68.5	7.53	29.4	5.55	1.05	4.88	0.71	3.97	0.75	2.19	0.34	2.38	0.37
G-s-3	2.68	294.1	4.53	18.6	25.6	54.2	6.23	24.4	4.74	0.89	4.31	0.64	3.93	0.75	2.14	0.34	2.27	0.36
G-s-6	2.32	317.3	5.59	23.4	32.0	68.4	8.07	31.0	6.06	1.11	5.48	0.84	4.86	0.94	2.69	0.41	2.84	0.41
G-s-18	4.30	317.3	5.47	17.1	35.6	75.1	8.68	32.9	6.24	1.10	5.55	0.81	4.54	0.81	2.39	0.37	2.60	0.41
G-b-4	3.37	425.7	5.04	19.7	30.9	67.7	7.70	29.4	5.65	1.05	4.94	0.76	4.30	0.80	2.40	0.36	2.48	0.38
G-b-7	2.33	255.4	4.51	17.5	26.6	55.6	6.35	24.5	4.71	0.86	4.19	0.63	3.64	0.71	2.12	0.32	2.27	0.35
G-b-10	4.97	317.3	5.44	16.8	28.8	61.8	7.03	28.2	5.43	1.00	5.01	0.74	4.05	0.78	2.34	0.36	2.48	0.38
G-f-11	3.17	263.2	6.18	16.4	30.3	64.2	7.19	28.7	5.50	0.98	4.94	0.71	4.01	0.77	2.28	0.35	2.40	0.36
B-c-12	0.70	162.5	3.77	14.1	23.6	48.9	5.75	22.0	4.47	0.79	3.27	0.50	2.78	0.54	1.63	0.26	1.72	0.25
B-c-13	0.74	162.5	3.69	13.0	21.8	46.1	5.35	20.6	4.27	0.77	3.09	0.48	2.58	0.50	1.55	0.25	1.68	0.25
B-c-18	0.75	185.8	4.07	14.4	23.8	47.7	5.33	20.2	4.20	0.77	3.22	0.50	2.66	0.53	1.72	0.27	1.81	0.29
B-s-14	0.78	216.7	4.33	14.9	26.3	52.6	6.24	21.9	4.77	0.86	3.77	0.56	2.89	0.54	1.79	0.30	1.95	0.31
B-s-16	0.80	193.5	4.12	14.9	27.1	55.7	6.41	23.6	4.60	0.76	3.85	0.58	3.04	0.56	1.71	0.26	1.91	0.30
B-s-19	0.76	162.5	3.98	14.1	23.6	50.4	5.37	22.3	4.83	0.80	3.36	0.53	2.93	0.55	1.66	0.28	1.79	0.28
B-b-15	0.66	154.8	3.58	12.8	22.8	46.6	5.52	20.1	3.82	0.66	3.30	0.48	2.61	0.45	1.49	0.25	1.59	0.25
B-b-17	0.67	147.1	3.51	11.8	21.0	45.4	5.24	18.9	3.85	0.67	3.02	0.44	2.51	0.45	1.38	0.21	1.54	0.26
B-b-20	0.78	193.5	4.06	14.6	26.8	56.9	6.42	26.1	4.88	0.82	3.87	0.56	3.33	0.62	1.82	0.27	1.90	0.31
B-b-21	0.83	286.4	4.09	13.9	26.1	55.4	6.44	24.0	4.54	0.80	3.61	0.56	2.91	0.58	1.64	0.25	1.81	0.29
B-f-22	0.69	170.3	3.69	13.0	24.5	51.8	5.80	22.5	4.32	0.73	3.70	0.51	2.82	0.50	1.49	0.23	1.58	0.28
Particle size fraction 4: 10–1 µm																		
G-c-1	4.6	1006.2	8.41	19.9	35.3	55.9	8.27	31.7	5.80	1.14	5.09	0.75	4.46	0.81	2.46	0.36	2.35	0.34
G-c-2	3.6	743.0	9.00	19.1	36.5	82.1	8.66	32.0	5.92	1.17	5.24	0.77	4.37	0.80	2.27	0.35	2.28	0.34
G-c-5	4.2	704.3	10.60	31.5	36.3	61.4	9.52	37.2	7.46	1.59	7.21	1.03	6.02	1.16	3.28	0.47	3.03	0.45
G-c-8	5.8	673.4	10.00	20.3	46.1	93.6	9.51	36.7	6.85	1.36	6.26	0.93	4.98	0.98	2.67	0.39	2.57	0.39
G-s-3	3.9	774.0	10.10	31.1	44.2	73.0	11.50	44.7	9.09	1.90	8.25	1.24	7.37	1.33	3.80	0.53	3.42	0.49
G-s-6	8.1	1006.2	10.90	35.9	40.7	66.0	10.70	44.0	8.74	1.84	8.43	1.27	7.13	1.34	3.90	0.55	3.52	0.53
G-s-18	10.8	743.0	10.40	21.5	41.3	92.0	9.87	39.6	7.71	1.55	6.89	1.07	5.87	1.01	3.06	0.40	2.67	0.38
G-b-4	3.9	928.8	9.81	31.2	29.6	67.3	10.20	40.9	8.24	1.70	7.32	1.11	6.28	1.19	3.41	0.48	3.15	0.45
G-b-7	4.2	712.1	9.78	30.4	43.7	71.8	11.10	44.2	8.85	1.87	8.20	1.19	6.73	1.25	3.48	0.51	3.26	0.49
G-b-10	6.3	626.9	10.40	31.1	43.9	103.7	11.10	45.9	9.44	1.99	9.17	1.31	7.25	1.40	3.99	0.57	3.60	0.54
G-f-11	8.9	766.3	12.20	27.1	43.6	71.3	11.30	45.7	9.00	1.91	8.04	1.16	6.54	1.22	3.42	0.49	3.21	0.46
B-c-12	2.1	379.3	8.68	17.7	31.3	69.4	7.51	28.7	5.72	1.25	4.21	0.65	3.56	0.71	2.04	0.32	2.17	0.31
B-c-13	2.0	387.0	8.25	17.3	30.8	63.0	7.17	27.9	5.34	1.09	4.13	0.62	3.41	0.70	1.99	0.31	2.06	0.28
B-c-18	2.1	541.8	8.99	18.9	35.6	78.0	8.05	31.7	6.39	1.22	4.95	0.73	4.47	0.76	2.13	0.34	2.28	0.35
B-s-14	1.9	657.9	8.06	15.9	30.1	64.3	6.88	24.6	4.84	0.95	4.00	0.60	3.16	0.55	1.84	0.28	1.85	0.29
B-s-16	2.1	588.2	8.97	17.4	34.3	76.1	8.03	28.6	5.64	1.15	4.64	0.69	3.65	0.69	1.97	0.31	2.07	0.33
B-s-19	2.4	425.7	10.10	20.5	35.6	85.8	7.99	32.0	6.37	1.30	4.83	0.75	4.30	0.80	2.28	0.35	2.23	0.35
B-b-15	2.1	479.9	9.29	19.7	37.2	77.5	8.66	31.6	6.09	1.32	5.01	0.78	3.96	0.76	2.30	0.34	2.27	0.36
B-b-17	2.0	441.2	9.13	20.4	35.2	77.6	7.92	30.3	6.09	1.24	4.77	0.70	3.81	0.72	2.07	0.33	2.18	0.33
B-b-20	2.3	611.5	8.73	21.9	37.0	85.8	8.93	35.9	7.38	1.31	5.55	0.81	4.71	0.85	2.46	0.36	2.36	0.37
B-b-21	2.4	1083.6	8.76	22.9	38.9	89.7	9.22	34.6	7.10	1.33	5.51	0.83	4.62	0.85	2.54	0.40	2.37	0.38
B-f-22	2.2	472.1	9.27	19.7	37.3	82.4	8.97	34.6	6.39	1.36	5.36	0.77	4.10	0.81	2.50	0.37	2.37	0.36
Particle size fraction 5: < 1 µm																		
G-c-1	7.1	5882.4	21.9	21.4	45.0	82.5	10.20	37.1	7.02	1.47	6.16	0.98	5.61	1.04	2.90	0.42	2.66	0.40
G-c-8	9.7	2554.2	22.6	17.5	38.4	95.3	8.73	33.3	5.83	1.24	5.40	0.82	4.61	0.81	2.30	0.35	2.21	0.35
G-s-3	6.8	2554.2	23.8	25.0	43.8	64.6	10.60	40.0	7.74	1.59	6.88	1.01	5.96	1.09	3.20	0.44	2.91	0.43
G-s-6	9.3	1702.8	19.5	31.6	44.7	67.0	11.10	44.3	8.45	1.76	7.53	1.13	6.36	1.18	3.39	0.46	3.08	0.45
G-s-9	9.2	1083.6	21.8	15.3	33.7	73.4	7.60	28.0	5.21	1.13	4.62	0.72	3.83	0.74	2.10	0.31	2.04	0.32
G-b-4	6.6	2709.0	21.0	22.1	39.7	81.8	9.55	36.9	6.97	1.46	6.10	0.88	5.16	0.96	2.74	0.38	2.60	0.37
G-b-7	12.8	2476.8	20.4	19.0	41.1	90.1	10.00	39.3	7.61	1.57	6.84	1.01	5.65	1.06	2.93	0.42	2.70	0.42
G-b-10	9.8	1315.8	25.2	29.6	47.5	74.4	12.40	48.4	9.42	2.04	8.52	1.27	7.14	1.30	3.71	0.53	3.51	0.48
B-c-12	8.4	2399.4	18.9	19.5	37.5	80.9	8.40	30.2	5.69	1.18	5.00	0.86	4.58	0.81	2.32	0.34	2.92	0.30
B-c-13	9.9	2709.0	21.1	20.3	37.3	78.7	8.49	30.8	6.18	1.30	5.07	0.77	4.62	0.90	2.71	0.35	2.11	0.31
B-c-18	17.5	5108.4	16.4	14.9	28.8	66.8	6.08	25.7	5.16	1.11	4.05	0.64	4.32	0.75	2.07	0.30	1.89	0.25
B-s-14	10.6	3715.2	15.1	14.2	27.2	58.6	5.73	21.8	4.44	0.93	3.62	0.54	3.19	0.63	1.82	0.27	1.59	0.28
B-s-16	8.4	4024.8	20.8	19.7	33.2	73.4	7.04	24.7	5.67	1.10	4.44	0.72	4.51	0.85	2.25	0.33	2.29	0.32
B-s-19	9.9	2167.2	20.2	15.2	29.3	63.7	5.84	23.8	4.72	0.96	3.39	0.53	3.21	0.67	1.77	0.30	1.88	0.26
B-b-15	9.1	4489.2	17.6	19.4	33.4	72.4	7.23	25.6	5.16	1.18	4.80	0.75	3.90	0.75	2.18	0.33	2.10	0.31
B-b-17	6.4	4334.4	20.2	22.2	34.9	78.8	7.90	30.6	6.16	1.31	5.03	0.83	4.61	1.03	2.69	0.37	2.24	0.32
B-b-20	7.4	6269.4	20.8	22.1	35.9	85.6	8.31	29.0	6.12	1.24	5.42	0.84	4.49	0.87	2.63	0.42	2.35	0.37
B-b-21	8.5	4721.4	20.7	25.7	40.1	92.8	9.32	38.8	7.60	1.54	6.33	1.00	5.94	1.20	2.99	0.45	2.64	0.45
B-f-22	12.6	4257.0	19.4	21.5	38.6	86.8	9.07	34.6	6.67	1.38	5.97	0.90	5.22	0.99	2.78	0.39	2.29	0.35
Methods used for determination of elements and lower limits of detection (LOD), µg/g																		
Method	AE*	AE	MS**	MS	MS	MS	MS	MS	MS	MS	MS	MS	MS	MS	MS	MS	MS	MS
LOD	0.00014	0.000012	0.14	0.0038	0.017	0.016	0.002	0.0069	0.0033	0.0011	0.0025	0.00051	0.0017	0.00067	0.0013	0.00049	0.0011	0.00058

*AE - ICP-AES.

** MS - ICP-MS.

## 2 Experimental design, materials, and methods

### 2.1 Dataset area and objects

The dataset area is located in the south-eastern part of the Smolensk-Moscow Upland, in the Middle Protva basin, 100 km to the southwest from Moscow near the border between the Moscow and Kaluga regions (Russia). It represents a marginal area of the Middle Pleistocene (MIS 6) glaciation and is located in a transition zone from mixed to deciduous forests. The dataset objects are a “V”-shaped gully and a small “U”-shaped dry valley (called balka in Russian) incised into the left side of the Protva river valley (Fig. 1).

The gully and the balka represent two common types of small erosional systems that are widespread in the dataset area. The gully is a smaller and younger Holocene landform [7]. It has a well-developed fan, a concave form of longitudinal profile and mostly “V”-shaped cross-sections with sharp edges and straight sides. It has very little sediment storages in its bottom (Fig. 2). The soils are formed on different parent materials since the system cuts through various lithologies including glaciofluvial sands (Fig. 2). Loamy deposits occupy its catchment but have limited exposures in the landform itself: Late Pleistocene loessial loams cover the headwaters and boulder clays are exposed in the gully lower reaches (Fig. 2). The soil cover of the gully catchment is dominated by soil formed under forest communities classified as Retisols according to IUSS Working Group WRB [8,9], but in the bottom and on the sides of the landform the forest soils have a weakly differentiated profile of Regosol [9]. The gully detrital fan is occupied by herbaceous meadow communities where Regosols with a relatively thick humus horizon are formed [8].

Another landform, the balka, is morphologically different and an older system whose formation was initiated in the Pleistocene [10]. The balka is incised mostly into loamy deposits, such as Late Pleistocene loessial loams and Middle Pleistocene glacial sediments. It has a smoothed longitudinal profile, a well-developed fan and “U”-shaped cross-sections. The bottom of the balka is covered by loamy sediments (Fig. 2) accumulated during the periods of low erosion [7], therefore the parent material for soil formation is more homogeneous than in the gully. The soils of the balka sides and bottom are Regosols [8,9] developing under grass and large shrub vegetation. The balka catchment area with Retisols [8] on loessial deposits was used as tillage and nowadays is occupied by grass vegetation. Thus, the gully and the balkа, having different morphology, belong to different lithological types, which allows one to evaluate the effect of parent lithology on particle size partitioning of REEs: the balka can be viewed as more homogeneous, monolithic, system with loamy deposits serving as parent material and gully belongs to a sandier and less homogeneous, heterolithic type.

### 2.2 Sampling procedure

Soil samples were taken of the top 10 cm at 22 locations (11 locations in each landform). The sample collection was performed along several cross-sections (transects) located in the upper, middle and lower reaches of the landforms and also along their bottoms (Fig 1a–d). Four sets of soil samples collected in each landform represent the upper soil horizons of (1) the landform sides, (2) the bottom and (3) the detrital fan and also (4) the adjacent catchment area considered as a source of solid matter.

### 2.3 Laboratory methods

The collected 22 bulk samples were air-dried, crushed to pass through a 1 mm sieve and analysed for organic carbon content, exchangeable soil acidity in 1 M KCl solution, and particle size analysis. The concentrations of total organic carbon (TOC) were determined using K_2_Cr_2_O_7_ wet-combustion method [2]. The particle size analysis was performed after pretreatment of the samples with sodium pyrophosphate [1] without H_2_O_2_ oxidation of organic matter. In physical fractionation the sand fractions were separated from the bulk soil samples by wet sieving while the silt fractions, as well as the clay fraction, were obtained by sedimentation and siphoning, during times determined by Stokes’ law. The boundaries between particle size classes were defined in accordance with the Russian conventional fraction groups [1]: coarse and medium sand (1000–250 µm), fine sand (250–50 µm), coarse silt (50–10 µm), medium and fine silt (10–1 µm), clay (<1 µm). In total 22 bulk samples of humus horizons and 100 samples of their individual fractions were analysed for the REEs’ contents. The concentrations of the REEs, Mn and Fe were determined by ICP-MS and ICP-AES after digestion of samples in a mixture of acids (NSAM-499-AES/MS method) on Elan-6100 and Optima-4300 DV spectrometers (Perkin Elmer Inc., USA).

## Appendix Supplementary materials

Image, application 1
